# Supplementary material for: Machine Learning Accelerates Crystallization for Structure Determination
Source: Angew Chem Int Ed Engl. 2026 May 4;65(25):e1218503. doi: 10.1002/anie.1218503 (PMC13266958; doi:10.1002/anie.1218503)

checkCIF/PLATON report

Structure factors have been supplied for datablock(s) lcz029

THIS REPORT IS FOR GUIDANCE ONLY. IF USED AS PART OF A REVIEW PROCEDURE FOR PUBLICATION, IT SHOULD NOT REPLACE THE EXPERTISE OF AN EXPERIENCED CRYSTALLOGRAPHIC REFEREE.

No syntax errors found. CIF dictionary Interpreting this report

Datablock: lcz029

Bond precision:	C-C = 0.0095 A		Wavelength=1.54184
Cell:	a=23.0450(3)	b=9.4650(1)	c=15.9513(2)
	alpha=90	beta=109.340(1)	gamma=90
Temperature:	100 K		

	Calculated	Reported
Volume	3282.98(7)	3282.97(7)
Space group	P 21/c	P 1 21/c 1
Hall group	-P 2ybc	-P 2ybc
Moiety formula	C25 H13 Ag3 F18 N6 O2	C15 H3 Ag3 F18 N6, C10 H10 O2
Sum formula	C25 H13 Ag3 F18 N6 O2	C25 H13 Ag3 F18 N6 O2
Mr	1095.03	1095.02
Dx,g cm-3	2.216	2.215
Z	4	4
Mu (mm-1)	15.531	15.531
F000	2096.0	2096.0
F000'	2106.24	
h,k,lmax	29,12,20	29,11,19
Nref	7049	6907
Tmin,Tmax	0.060,0.045	0.521,1.000
Tmin'	0.004	

Correction method= # Reported T Limits: Tmin=0.521 Tmax=1.000
AbsCorr = MULTI-SCAN

Data completeness= 0.980 Theta(max)= 78.377

R(reflections)= 0.0608(6600) wR2(reflections)= 0.1663(6907)

S = 1.043 Npar= 488

The following ALERTS were generated. Each ALERT has the format
test-name_ALERT_alert-type_alert-level.
Click on the hyperlinks for more details of the test.

Alert level C

```
PLAT042_ALERT_1_C Calc. and Reported MoietyFormula Strings  Differ     Please Check
              Calc: C25 H13 Ag3 F18 N6 O2
              Rep.: C15 H3 Ag3 F18 N6, C10 H10 O2
PLAT213_ALERT_2_C Atom F00J           has ADP max/min Ratio .....         3.1 prolat
PLAT242_ALERT_2_C Low    'MainMol' Ueq as Compared to Neighbors of       C014 Check
PLAT242_ALERT_2_C Low    'MainMol' Ueq as Compared to Neighbors of       C016 Check
PLAT342_ALERT_3_C Low Bond Precision on  C-C Bonds ...............     0.0095 Ang.
PLAT906_ALERT_3_C Large K Value in the Analysis of Variance ......      2.891 Check
PLAT911_ALERT_3_C Missing FCF Refl Between Thmin & STh/L=    0.600          7 Report
                0  0  2,  -1  1  4,  10  0 14, -17  3 17, -13  0 18,  -5  0 18,
               -4  0 18,
PLAT971_ALERT_2_C Check Calcd Resid. Dens.  0.99Ang From Ag01           1.76 eA-3
PLAT971_ALERT_2_C Check Calcd Resid. Dens.  1.05Ang From N00S           1.71 eA-3
PLAT971_ALERT_2_C Check Calcd Resid. Dens.  1.12Ang From Ag02           1.63 eA-3
PLAT972_ALERT_2_C Check Calcd Resid. Dens.  0.82Ang From Ag02          -2.37 eA-3
PLAT972_ALERT_2_C Check Calcd Resid. Dens.  0.72Ang From Ag03          -2.22 eA-3
PLAT972_ALERT_2_C Check Calcd Resid. Dens.  0.69Ang From Ag02          -2.16 eA-3
PLAT972_ALERT_2_C Check Calcd Resid. Dens.  0.75Ang From Ag01          -2.14 eA-3
PLAT972_ALERT_2_C Check Calcd Resid. Dens.  0.73Ang From Ag01          -2.14 eA-3
PLAT972_ALERT_2_C Check Calcd Resid. Dens.  0.74Ang From Ag03          -2.06 eA-3
PLAT972_ALERT_2_C Check Calcd Resid. Dens.  0.85Ang From Ag03          -1.72 eA-3
PLAT972_ALERT_2_C Check Calcd Resid. Dens.  0.83Ang From Ag01          -1.60 eA-3
PLAT972_ALERT_2_C Check Calcd Resid. Dens.  0.89Ang From Ag02          -1.59 eA-3
PLAT976_ALERT_2_C Check Calcd Resid. Dens.  0.89Ang From O00A    .      -0.86 eA-3
PLAT976_ALERT_2_C Check Calcd Resid. Dens.  1.04Ang From O00A    .      -0.86 eA-3
PLAT976_ALERT_2_C Check Calcd Resid. Dens.  0.73Ang From O00A    .      -0.75 eA-3
```

Alert level G

```
PLAT072_ALERT_2_G SHELXL First  Parameter in WGHT  Unusually Large       0.12 Report
PLAT083_ALERT_2_G SHELXL Second Parameter in WGHT  Unusually Large      16.63 Why ?
PLAT242_ALERT_2_G Low    'MainMol' Ueq as Compared to Neighbors of       C00V Check
PLAT242_ALERT_2_G Low    'MainMol' Ueq as Compared to Neighbors of       C012 Check
PLAT242_ALERT_2_G Low    'MainMol' Ueq as Compared to Neighbors of       C013 Check
PLAT434_ALERT_2_G Short Inter HL..HL Contact F005     ..F00H     .       2.81 Ang.
                                            1-x,1/2+y,1/2-z  =      2_655 Check
PLAT434_ALERT_2_G Short Inter HL..HL Contact F009     ..F00Q     .       2.84 Ang.
                                              x,1/2-y,1/2+z  =      4_566 Check
PLAT434_ALERT_2_G Short Inter HL..HL Contact F00G     ..F00Q     .       2.76 Ang.
                                              x,1/2-y,1/2+z  =      4_566 Check
PLAT720_ALERT_4_G Number of Unusual/Non-Standard Labels ..........          67 Note
              Ag01    Ag02    Ag03    F004    F005    F006    F007    F008
```

```
              F009    O00A    F00B    F00C    F00D    F00E    F00F    F00G
              F00H    F00I    F00J    F00K    N00L    F00M    N00N    N00O
              N00P    F00Q    N00R    N00S    O00T    C00U    H00U    C00V
              C00W    H00W    C00X    C00Y    C00Z    C010    C011    C012
              C013    C014    C015    C016    C017    C018    H018    C019
              H019    C01A    C01B    C01C    C01D    C01E    H01A    H01B
              C01F    H01C    H01D    C01G    H01E    H01F    H01G    C01H
              H01H    C01I    H01I
PLAT883_ALERT_1_G Absent Datum for _atom_sites_solution_primary ..     Please Do !
PLAT910_ALERT_3_G Missing FCF Reflection(s) Below Theta(Min)[Deg]=       4.07 Note
                1  0  0,
PLAT912_ALERT_4_G Missing # of FCF Reflections Above STh/L=  0.600        113 Note
PLAT933_ALERT_2_G Number of HKL-OMIT Records in Embedded .res File          2 Note
              -18  1 18, -13  0 18,
PLAT969_ALERT_5_G The 'Henn et al.' R-Factor-gap value ...........      5.247 Note
              Predicted wR2: Based on SigI**2  3.17 or SHELX Weight 15.95
PLAT978_ALERT_2_G Number C-C Bonds with Positive Residual Density.          0 Info
```

```
   0 ALERT level A = Most likely a serious problem - resolve or explain
   0 ALERT level B = A potentially serious problem, consider carefully
  22 ALERT level C = Check. Ensure it is not caused by an omission or oversight
  15 ALERT level G = General information/check it is not something unexpected

   2 ALERT type 1 CIF construction/syntax error, inconsistent or missing data
  28 ALERT type 2 Indicator that the structure model may be wrong or deficient
   4 ALERT type 3 Indicator that the structure quality may be low
   2 ALERT type 4 Improvement, methodology, query or suggestion
   1 ALERT type 5 Informative message, check
```

Validation response form

Please find below a validation response form (VRF) that can be filled in and pasted into your CIF.

```
# start Validation Reply Form
_vrf_PLAT042_lcz029
;
PROBLEM: Calc. and Reported MoietyFormula Strings  Differ     Please Check
RESPONSE: ...
;
_vrf_PLAT213_lcz029
;
PROBLEM: Atom F00J            has ADP max/min Ratio .....        3.1 prolat
RESPONSE: ...
;
_vrf_PLAT242_lcz029
;
PROBLEM: Low    'MainMol' Ueq as Compared to Neighbors of       C014 Check
RESPONSE: ...
;
_vrf_PLAT342_lcz029
;
PROBLEM: Low Bond Precision on  C-C Bonds ..............     0.0095 Ang.
RESPONSE: ...
;
_vrf_PLAT906_lcz029
```

```
;
PROBLEM: Large K Value in the Analysis of Variance ......      2.891 Check
RESPONSE: ...
;
_vrf_PLAT911_lcz029
;
PROBLEM: Missing FCF Refl Between Thmin & STh/L=    0.600          7 Report
RESPONSE: ...
;
_vrf_PLAT971_lcz029
;
PROBLEM: Check Calcd Resid. Dens.  0.99Ang From Ag01            1.76 eA-3
RESPONSE: ...
;
_vrf_PLAT972_lcz029
;
PROBLEM: Check Calcd Resid. Dens.  0.82Ang From Ag02           -2.37 eA-3
RESPONSE: ...
;
_vrf_PLAT976_lcz029
;
PROBLEM: Check Calcd Resid. Dens.  0.89Ang From O00A    .      -0.86 eA-3
RESPONSE: ...
;
# end Validation Reply Form
```

It is advisable to attempt to resolve as many as possible of the alerts in all categories. Often the minor alerts point to easily fixed oversights, errors and omissions in your CIF or refinement strategy, so attention to these fine details can be worthwhile. It is up to the individual to critically assess their own results and, if necessary, seek expert advice.

PLATON version of 17/09/2025; check.def file version of 17/09/2025

---

duplicate check

No duplication found

Datablock lcz029 - ellipsoid plot

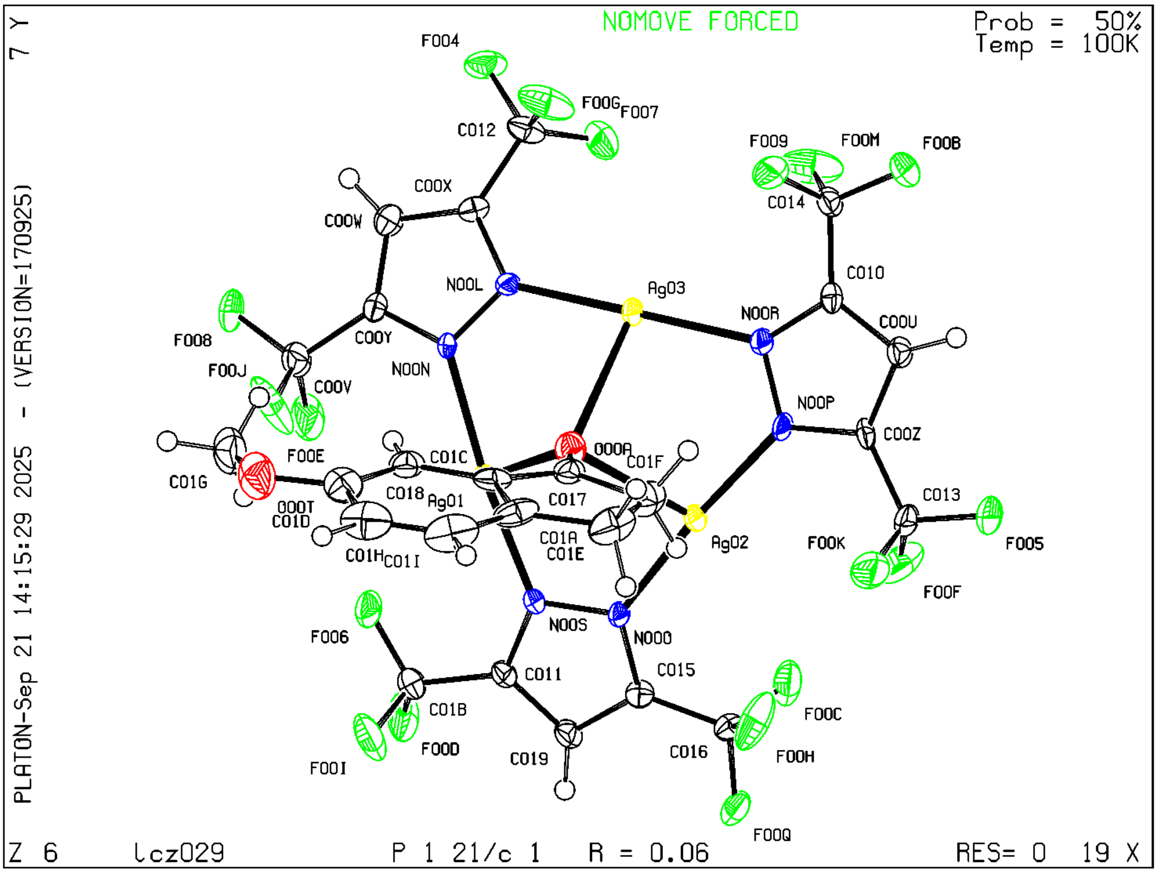

Supplement: Supplementary file 2 — Supporting File 2: anie72462‐sup‐0002‐DataFile.zip. [file ANIE-65-e1218503-s002.zip › supplementary_materials_crystallographic_data submit.zip!/cif_and_checkcif(1-114)/Ag3Pz3_29/Ag3Pz3_29_checkcif.pdf]
